# Concomitant primary breast carcinoma and primary choroidal melanoma: a case report

**DOI:** 10.1186/1752-1947-2-88

**Published:** 2008-03-19

**Authors:** Hari Jayaram, Asifa Shaikh, Sundeep Kheterpal

**Affiliations:** 1 Prince Charles Eye Unit, King Edward VII Hospital, St Leonard's Road, Windsor, SL4 3DP, UK

## Abstract

**Introduction:**

Choroidal melanoma and choroidal metastasis are distinct pathological entities with very different treatments and prognoses. They may be difficult to distinguish to the untrained observer.

**Case presentation:**

A case of concomitant choroidal melanoma in a woman with primary breast carcinoma is described. The choroidal lesion was thought initially to be a metastasis, and treated with external beam radiotherapy. The tumour did not regress but remained stable in size for a period of three years. Following referral to an ophthalmologist, the diagnosis was revised after re-evaluation of the clinical, ultrasonographic and angiographic findings.

**Conclusion:**

Although metastases are the most common ocular tumour, a differential diagnosis of a concurrent primary ocular malignancy should always be considered, even in patients with known malignant disease. Thorough ophthalmic evaluation is important, as multiple primary malignancies may occur concomitantly. The prognostic and therapeutic implications of accurate diagnosis by an ophthalmologist are of profound significance to affected patients and their families.

## Introduction

Choroidal melanoma and choroidal metastasis are distinct pathological entities with very different treatments and prognoses. They may be difficult to distinguish to the untrained observer. A case of concomitant choroidal melanoma in a woman with primary breast carcinoma is described. The choroidal lesion was thought initially to be a metastasis, and treated with external beam radiotherapy. The tumour did not regress but remained stable in size for a period of three years. Following referral to an ophthalmologist, the diagnosis was revised after re-evaluation of the clinical, ultrasonographic and angiographic findings.

## Case presentation

A 76 year old woman underwent mastectomy for a primary breast malignancy, shown histologically to be a low grade ductal adenocarcinoma (stage T_1 _N_0_). Three months after surgery, she complained of visual deterioration in her right eye. A lesion was identified on fundoscopy by the treating oncologists, and a presumptive diagnosis of choroidal metastasis from the breast malignancy was made without ophthalmic consultation. Palliative external beam radiotherapy (EBRT) (20 Gy total) was administered to the right orbit in five daily fractions. The patient was kept under regular review by her oncologist and remained stable with no enlargement of the lesion reported on serial magnetic resonance imaging.

**Figure 1:**
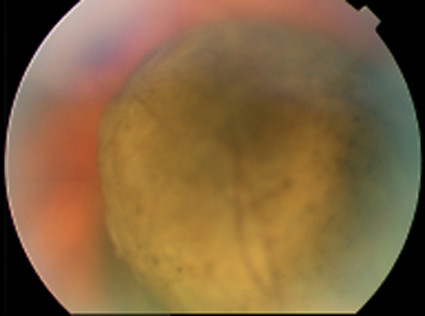
A large mushroom shaped lesion with some intrinsic pigmentation seen on examination of the right fundus.

**Figure 2:**
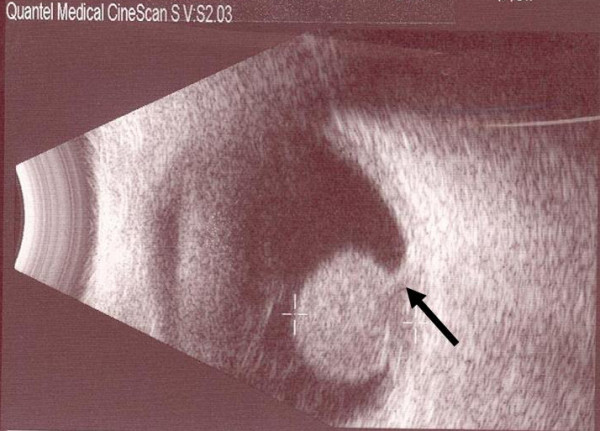
**B-scan ultrasound of the right eye showing the tumour.** The arrow points to excavation of the choroid by the invading tissue.

**Figure 3:**
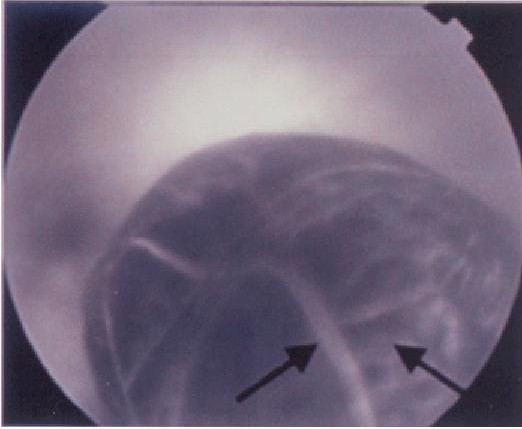
Fluorescein angiography of the right eye showing a "double circulation" (arrows) associated with the tumour.

Eighteen months after radiotherapy the patient was referred to our ophthalmic service due to failing vision in the right eye. Corrected visual acuity was 6/18 in the affected eye. Dilated examination using a slit lamp revealed an 11 × 10 mm elevated choroidal mass in the peripheral fundus, mainly yellow in color with some intrinsic pigmentation (Figure 1) and with no associated sub-retinal fluid. B-scan ultrasonography showed a mushroom shaped lesion, choroidal excavation due to extension through Bruch's membrane and low internal reflectivity (Figure 2). Fluorescein angiography demonstrated a "double circulation" (Figure 3) with intrinsic vasculature seen within the tumour, and the larger normal retinal vessels seen more superficially. Examination of the left eye was unremarkable.

A revised diagnosis was made of a primary choroidal melanoma, partially treated by radiotherapy, in the presence of a concomitant primary breast malignancy. Magnetic resonance imaging of the brain, chest radiographs and liver function tests demonstrated no evidence of metastatic disease. The patient declined further intervention initially and conservative management was initiated.

Three years later, growth of the lesion was observed and the patient was referred to a regional ocular oncology service. Enucleation was performed, over four years after the initial observation of the ocular lesion, confirming the diagnosis of choroidal melanoma. To date, five years since initial detection of the lesion, the patient remains well with no evidence of metastatic melanoma.

## Discussion

Metastatic disease is the most common ocular malignancy. Shields *et al *performed a retrospective survey of 520 eyes with uveal metastases of which 88% were within the choroid [1]. 66% of these cases had a known primary carcinoma, the most common sources being breast (47%) followed by lung (21%). Of the remainder, a primary malignancy was identified in only 50% of cases. Metastatic lesions in the choroid were typically yellow in colour, plateau shaped, associated with sub-retinal fluid and had a mean thickness of 3 mm.

Prospective follow up of patients enrolled in the Collaborative Ocular Melanoma Study (COMS) Group found that 7.7% of patients were diagnosed with a secondary primary malignancy over five years of follow up, with prostate (23%) and breast (17%) being most commonly reported [2].

Sobttka *et al *examined B-scan ultrasonographic findings in order to distinguish metastases in the choroid from primary malignant melanoma [3]. Choroidal excavation, low internal reflectivity and a high height:base ratio were considered to be virtually pathognomonic for choroidal melanoma. However "mushroom shaped" choroidal metastases have been reported [4,5], although these showed higher internal reflectivity on ultrasonography.

Studies of patients with choroidal metastases from primary breast carcinoma have reported a mean life expectancy of nine months following ocular diagnosis [6,7]. It is important to note that metastases exhibited bilaterality in 40% of cases and tended to follow pulmonary dissemination and to occur with or before central nervous system involvement [7].

The prolonged survival of this patient following detection of the choroidal tumour and the absence of metastatic disease at other sites further indicates that the ocular lesion was unlikely to be a metastasis, and was in fact a primary malignant melanoma whose growth had been arrested by radiotherapy. In addition the intrinsic or "double" circulation seen on fluorescein angiography in this case would be very atypical for a metastasis (Figure 3).

Treatment options for a primary choroidal melanoma as in this case would include brachytherapy, proton beam radiotherapy or enucleation, whereas breast metastases are often reviewed following systemic chemotherapy or external beam radiotherapy.

20 Gy of EBRT would be regarded as a sub-optimal treatment dose for choroidal melanoma. The 5 year melanoma-specific mortality for adequately treated medium sized choroidal melanoma has been reported at 10% by the COMS group [8] with undetectable micrometastases thought to occur early in the disease course, often before conservative treatment of the primary tumour [9]. The patient declined further active treatment initially, opting for a conservative approach, although definitive treatment was agreed upon following the detection of further growth of the melanoma.

## Conclusion

Although metastases are the most common ocular tumour, a differential diagnosis of a concurrent primary ocular malignancy should always be considered, even in patients with known malignant disease. Thorough ophthalmic evaluation is important, as multiple primary malignancies may occur concomitantly [10]. This is particularly important in the absence of either pulmonary or central nervous system involvement as metastatic ocular involvement usually occurs at an advanced stage. The prognostic and therapeutic implications of accurate diagnosis by an ophthalmologist are of profound significance to affected patients and their families.

## Competing interests

The author(s) declare that they have no competing interests.

## Authors' contributions

SK was in charge of the overall care of the patient, with HJ and AS involved in follow up care. HJ researched the literature and prepared the manuscript with critical review from AS and SK. All three authors read and approved the final manuscript.

## Consent

Written informed consent was obtained from the patient for publication of this case report and accompanying images.
